# Pasteurella multocida Infection Presented With Frequent Diarrhea in the Cirrhotic Patient

**DOI:** 10.7759/cureus.18183

**Published:** 2021-09-22

**Authors:** Hiroki Okumura, Sho Nishiguchi

**Affiliations:** 1 General Internal Medicine, Shonan Kamakura General Hospital, Kamakura, JPN

**Keywords:** bacterial translocation, liver cirrhosis, bacteremia, symptoms of enteritis, pasteurella multocida

## Abstract

A 49-year-old woman with alcoholic cirrhosis who owned a pet cat was brought to the hospital with frequent diarrhea every 30 min for two days. She was treated intensively for septic shock; however, she died on the third day. *Pasteurella multocida* is known to cause soft tissue infections; however, in immunocompromised individuals, it can cause severe invasive infections. Physicians should consider *P. multocida* infection when a patient with liver cirrhosis presents in shock following symptoms of enteritis. Clinical decisions should be made considering that this infection is associated with a high mortality rate and rapid deterioration.

## Introduction

*Pasteurella multocida* infection has been reported to cause serious infections in immunocompromised patients, including those with liver cirrhosis [1,2]. In addition to skin and soft tissue infections and pneumonia, which are relatively frequent, there have been reports of peritonitis [3], meningitis [4], and endocarditis [5]; however, there was only one report of *Pasteurella* bacteremia preceded by symptoms of enteritis [6]. In this study, we report the second case of *P. multocida *bacteremia preceded by frequent diarrhea.

## Case presentation

A 49-year-old woman presented with diarrhea approximately once every 30 min for two days prior to her visit. On arrival, she had a fever of 39.0°C with chills. The patient had persistent diarrhea and fever and had trouble moving; hence she required emergency assistance and was brought to our hospital. She was a current smoker, who smoked 10 cigarettes/day for 29 years and consumed 1,500 mL of cocktails per day. She owned a pet cat, and the cat licked her body surface regularly. She had a history of depression; however, she had not consulted a doctor for five years. She had no other medical history and was not taking any medications, including any over-the-counter medications.

Her height and body weight were 162 cm and 60 kg, respectively. On admission, consciousness was assessed using the Glasgow Coma Scale (score, 14) (E4V4M6), her body temperature was 38.0℃, pulse was 132 beats/min, blood pressure was 88/51 mmHg, respiratory rate was 24 breaths/min, and the peripheral arterial oxygen saturation was 94% (nasal cannula, 1 L). Physical examination revealed livedo reticularis on the lower limbs. There was no site of soft tissue infection with redness, heat, or swelling throughout the body. The blood tests that were performed are shown in Table 1. Arterial blood gas analysis showed mixed alkalosis and hyperlactatemia (serum lactate, 8.4 mg/dL). Conventional abdominal computed tomography revealed mild enlargement and a diffuse low-density area of the liver.

**Table 1:** Laboratory results on admission PT: prothrombin time, APTT: activated partial thromboplastin time, FDP: fibrinogen degradation products, AST: aspartate aminotransferase, ALT: alanine aminotransferase, LDH: lactate dehydrogenase, γGTP: gamma-glutamyl transpeptidase, BUN: Blood urea nitrogen, CRP: c-reactive protein, PaO_2_: arterial partial pressure of oxygen, PaCO_2_: arterial partial pressure of carbon dioxide

Parameter	Value	Units
White blood cell	58	×10²/μL
Hemoglobin	10.8	g/dL
Platelets	6	×10⁴/μL
PT-%	79.9	%
APTT	32.6	sec
Fibrinogen	309	mg/dL
FDP	43.8	μg/mL
AT-Ⅲ	62.5	%
Total bilirubin	3.8	mg/dL
Direct bilirubin	2.1	mg/dL
AST	706	U/L
ALT	105	U/L
LDH	1063	U/L
ALP	333	U/L
γGTP	689	U/L
Total protein	6.1	g/dL
Albumin	3.1	g/dL
BUN	5.6	mg/dL
Creatinine	0.6	mg/dL
Uric acid	8.3	mg/dL
Na	136	mEq/L
K	1.9	mEq/L
Cl	77	mEq/L
Mg	0.7	mEq/L
Glucose	75	mg/dL
CRP	9.1	mg/dL
Hemoglobin A1c	4.6	%
Procalcitonin	5	ng/mL
Arterial blood gas analysis		nasal cannula 1L
pH	7.66	
PaO_2_	62	Torr
PaCO_2_	29	Torr
Bicarbonate	32	mEq/L
Lactate	8.4	mg/dL

**Figure 1:**
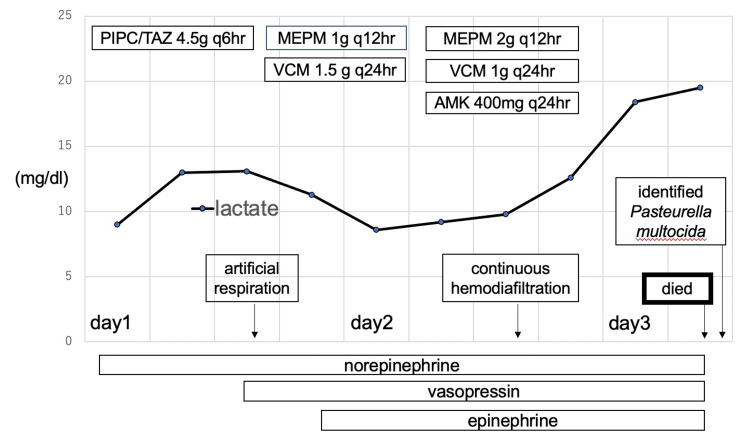
Clinical course of the patient PIPC/TAZ: piperacillin/tazobactam, MEPM: meropenem, VCM: vancomycin, AMK: amikacin

The patient was admitted to the intensive care unit in a state of septic shock with alcoholic cirrhosis, hypokalemia (serum potassium, 1.9 mEq/L) with frequent diarrhea, and disseminated intravascular coagulation syndrome. The clinical course of the patients is shown in Figure 1. Blood, urine, and spinal fluid specimens were cultured, and piperacillin-tazobactam (4.5 g) was administered as an empiric therapy with the initiation of potassium replacement through a central vein. On the same day, the antibacterial agents were changed to meropenem (1 g every 12 h) and vancomycin (1.5 g every 24 h) due to deteriorating clinical conditions. Artificial respiration was started on the same day as the patient's respiratory condition worsened. After admission, she did not have frequent diarrhea. On the second day of admission, the patient’s renal function (serum creatinine, 2.6 mg/dL) and liver function (prothrombin time, 35.6%; total bilirubin, 6.0 mg/dL) worsened, thereby requiring continuous hemodiafiltration (CHDF), plasma exchange therapy, and coagulation factor replacement. In addition, venoglobulin and amikacin 400 mg were administered for sepsis. The dosage of meropenem was changed to a severe dose of 2 g every 12 h considering the worsening clinical condition and the dosage of vancomycin was changed to 1 g every 24 h as the renal function worsened and CHDF was initiated. However, metabolic acidosis and hyperlactatemia progressed, and the patient died in the early hours of the third sick day. On the third day of admission, blood culture yielded* P. multocida*, which was susceptible to piperacillin-tazobactam and meropenem.

## Discussion

*P. multocida* is a commensal anaerobic Gram-negative coccobacillus that constitutes the bacterial flora of the upper respiratory tract and gastrointestinal tract of many animals [7]. *Pasteurella* is endemic to the oral cavity of cats and dogs and is a zoonotic agent [1]. Most *P. multocida* infections are skin and soft tissue infections, which in severe cases can lead to abscess formation, osteomyelitis, and septic arthritis [1,8]. Pneumonia and other respiratory tract infections are the second most common site of infection and are more likely to occur in patients with chronic obstructive pulmonary disease and other chronic respiratory diseases as comorbidities [8]. Peritonitis, meningitis, and pericarditis can also occur in immunocompromised patients with conditions such as cirrhosis, advanced age, chronic renal failure, malignancy, and diabetes mellitus [3,4,8]. However, there was only one report of bacteremia triggered by symptoms of enteritis such as frequent diarrhea [6].

In a review of 119 cases of *P. multocida* bacteremia [8], 80/119 patients had significant comorbidities such as cirrhosis, immunosuppressive therapy, and malignancy, with cirrhosis being the most common comorbidity. Only the presence of comorbidities was associated with mortality in the multivariate analysis. The mortality rate of *P. multocida* bacteremia was 31.1% [8]. Among the case reports, there are many cases of death occurring within a few days. The patient, in this case, owned a pet cat; however, no obvious bite wound was observed. The patient died on the third sick day because of septic shock that had a rapid course.

The patient had a history of alcoholism and was diagnosed with alcoholic liver cirrhosis based on blood tests and imaging studies. Cirrhotic patients are prone to bacteremia caused by bacterial translocation due to altered mononuclear phagocyte function and the presence of portosystemic shunt observed in cirrhosis [2]. In addition, in the basic experiment with mouse models of cirrhosis, it has been reported that *Pasteurella*-induced bacterial translocation is likely to occur [9]. Damage to the intestinal mucosa due to symptoms of enteritis is also known to cause bacterial translocation [10]. In this case, it is assumed that damage to the intestinal mucosa caused by alcoholic liver cirrhosis and diarrhea led to bacterial translocation and bacteremia.

## Conclusions

In patients with septic shock due to liver cirrhosis and preceding symptoms of enteritis, it is necessary to check for a history of pet ownership considering infection with *P. multocida*. The mortality of *P. multocida* infection in compromised patients, including those with alcoholic liver cirrhosis, is high. We suggest physicians should make clinical decisions considering that the patient's general condition may deteriorate rapidly.
